# Effects of neprilysin and neprilysin inhibitors on glucose homeostasis: Controversial points and a promising arena

**DOI:** 10.1111/1753-0407.13389

**Published:** 2023-04-19

**Authors:** Faisal Holil AlAnazi, Hayder M. Al‐kuraishy, Ali I. Al‐Gareeb, Athanasios Alexiou, Marios Papadakis, Hanan A. Ogaly, Yousef Abud Alanazi, Hebatallah M. Saad, Gaber El‐Saber Batiha

**Affiliations:** ^1^ Department of Medicine, College of Medicine Majmaah University Majmaah Saudi Arabia; ^2^ Department of Clinical Pharmacology and Medicine, College of Medicine ALmustansiriyia University Baghdad Iraq; ^3^ Department of Science and Engineering Novel Global Community Educational Foundation Hebersham New South Wales Australia; ^4^ AFNP Med Wien Austria; ^5^ Department of Surgery II University Hospital Witten‐Herdecke, University of Witten‐Herdecke Wuppertal Germany; ^6^ Chemistry Department, College of Science King Khalid University Abha Saudi Arabia; ^7^ Department of Pediatrics, College of Medicine Majmaah University Majmaah Saudi Arabia; ^8^ Department of Pathology, Faculty of Veterinary Medicine Matrouh University Marsa Matruh Egypt; ^9^ Department of Pharmacology and Therapeutics, Faculty of Veterinary Medicine Damanhour University Damanhour Egypt

**Keywords:** neprilysin, sacubitril, type 2 diabetes mellitus, 脑啡肽酶, 脑啡肽酶抑制剂, 2型糖尿病

## Abstract

Neprilysin (NEP) is a transmembrane zinc‐dependent metalloproteinase that inactivates various peptide hormones including glucagon‐like peptide 1 (GLP‐1). NEP inhibitors may be effective in the management of type 2 diabetes mellitus (T2DM) by increasing the circulating level of GLP‐1. However, acute‐effect NEP inhibitors may lead to detrimental effects by increasing blood glucose independent of GLP‐1. These findings suggest a controversial point regarding the potential role of NEP inhibitors on glucose homeostasis in T2DM patients. Therefore, this perspective aimed to clarify the controversial points concerning the role of NEP inhibitors on glucose homeostasis in T2DM. NEP inhibitors may lead to beneficial effects by inhibition of NEP, which is involved in the impairment of glucose homeostasis through modulation of insulin resistance. NEP increases dipeptidyl peptidase‐4 (DPP4) activity and contributes to increasing active GLP‐1 proteolysis so NEP inhibitors may improve glycemic control through increasing endogenous GLP‐1 activity and reduction of DPP4 activity. Thus, NEP inhibitors could be effective alone or in combination with antidiabetic agents in treating T2DM patients. However, long‐term and short‐term effects of NEP inhibitors may lead to a detrimental effect on insulin sensitivity and glucose homeostasis through different mechanisms including augmentation of substrates and pancreatic amyloid deposition. These findings are confirmed in animal but not in humans. In conclusion, NEP inhibitors produce beneficial rather than detrimental effects on glucose homeostasis and insulin sensitivity in humans though most of the detrimental effects of NEP inhibitors are confirmed in animal studies.

## Graphical abstract

**Highlights**

- Neprilysin (NEP) inhibitors may lead to beneficial effects through inhibition of NEP, which involves impairment of glucose homeostasis through modulation of insulin resistance and the development of pancreatic β cell dysfunction as observed in type 2 diabetes mellitus.
- NEP increases dipeptidyl peptidase‐4 (DPP4) activity and contributes to increasing active glucagon‐like peptide‐1 (GLP‐1) proteolysis so NEP inhibitors may improve glycemic control through increasing endogenous GLP‐1 activity and reduction of DPP4 activity.
- NEP inhibitors have beneficial effects on glucose homeostasis and insulin sensitivity by increasing oxytocin and various substrates including GLP‐1.
- NEP inhibitors may lead to a detrimental effect on insulin sensitivity and glucose homeostasis as well as pancreatic β cell dysfunction through different mechanisms including augmentation of some substrates and pancreatic amyloid deposition.

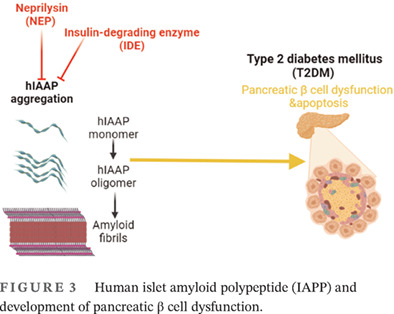

## 摘要

脑啡肽酶(NEP)是一种跨膜锌依赖性金属蛋白酶，可使包括胰高血糖素样肽1 (GLP‐1)在内的多种肽类激素失活。NEP抑制剂可能通过提高循环GLP‐1水平来有效治疗2型糖尿病(T2DM)。然而，NEP抑制剂的急性效应可能会造成血糖升高，产生有害作用，且与GLP‐1无关。这些发现提示NEP抑制剂对T2DM患者血糖稳态的作用尚存有争议。本视角旨在阐明NEP抑制剂对T2DM糖稳态作用的争议点。NEP通过调节胰岛素抵抗(IR)参与糖稳态受损，NEP抑制剂可能通过抑制NEP发挥有益作用。NEP增加二肽基肽酶‐4 (DPP4)活性并有助于增加GLP‐1蛋白水解活性，而NEP抑制剂可能通过增加内源性GLP‐1活性和降低DPP4活性来改善血糖控制。因此，NEP抑制剂可单独或联合降糖药物治疗T2DM患者。然而，NEP抑制剂的长期和短期作用机制可能不同，包括增强底物和胰腺淀粉样蛋白沉积，导致对胰岛素敏感性和葡萄糖稳态的有害影响。这些发现在动物中得到证实，但在人类中未得到证实。综上所述，NEP抑制剂对人体葡萄糖稳态和胰岛素敏感性产生有益而非有害的影响，尽管大多数有害影响已在动物研究中得到证实。

## 1 INTRODUCTION

**FIGURE 1:**
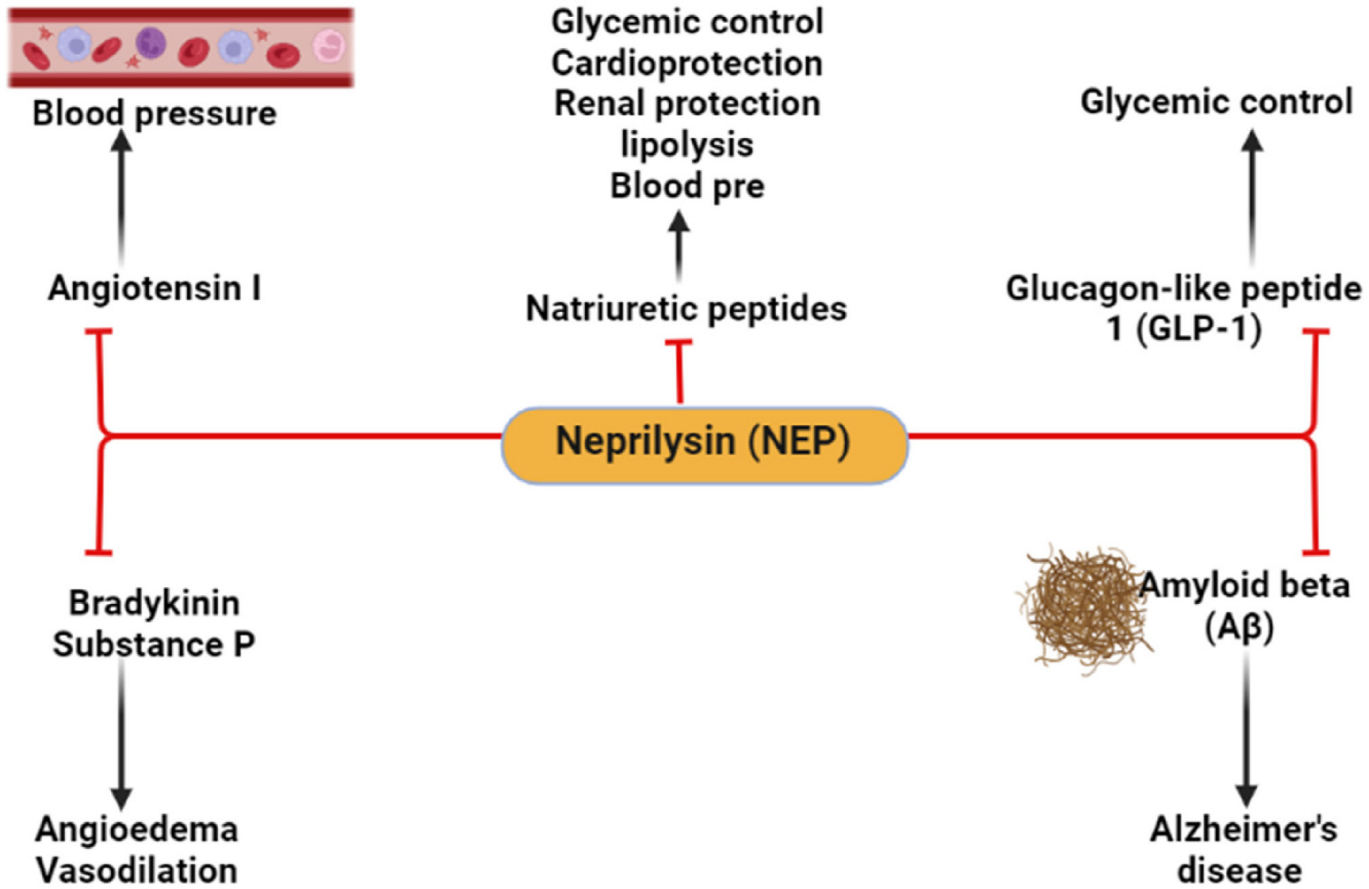
The physiological role of neprilysin (NEP) “Created with BioRender.com”.

Neprilysin (NEP) is also known as cluster of differentiation 10, enkephalinase, neutral endopeptidase, membrane metalloendopeptidase, and common acute lymphoblastic leukemia antigen (CALLA).^1^ NEP is a transmembrane zinc‐dependent metalloproteinase that inactivates various peptide hormones including natriuretic peptides, bradykinin, oxytocin, neurotensin, substance P, enkephalins, angiotensin II (AngII), endothelin 1 (ET‐1), and glucagon‐like peptide 1 (GLP‐1). NEP is also involved in the degradation of amyloid beta (Aβ) and could be effective in the prevention development of Alzheimer's disease (AD)^2^, ^3^, ^4^ (Figure 1).

NEP is widely expressed on the surface of endothelial cells, neutrophils, and fibroblasts and various tissues including the brain, kidneys, lungs, testes, gastrointestinal tract, and heart.^5^ Soluble NEP is found in the blood, which reflects the concentration and activity of membrane‐bound NEP.^6^ Indeed, NEP is mainly involved in neurohumoral activation and regulation of the sympathetic nervous system and renin‐angiotensin system (RAS) in different diseases including heart failure.^2^, ^4^, ^7^ Higher expression of NEP is associated with poor prognostic outcomes in patients with heart failure.^8^ However, in the cohort and mixed cohort studies, NEP level was of no clinical value in the prognosis of heart failure.^9^, ^10^

**FIGURE 2:**
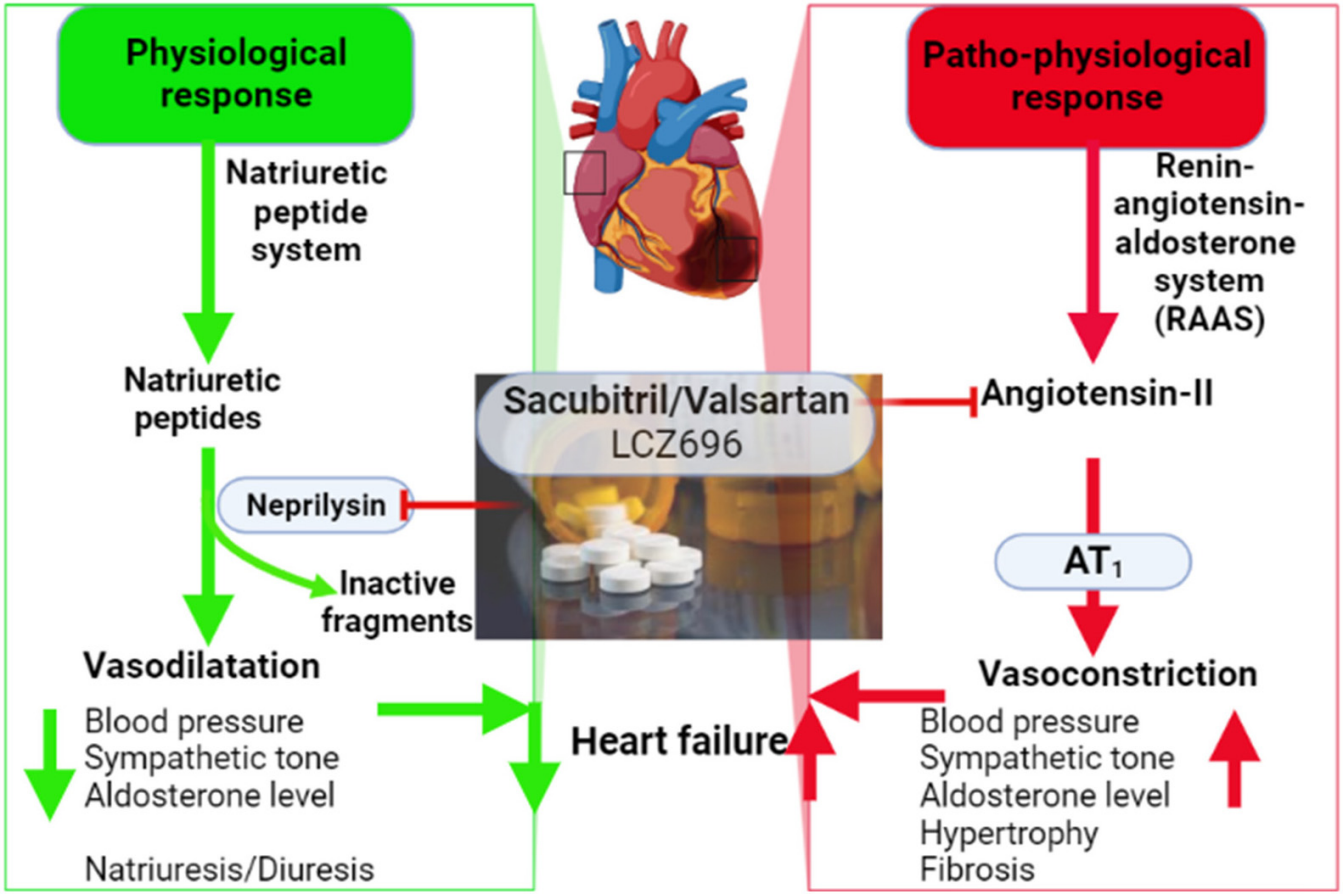
Neprilysin (NEP) inhibitors in the management of heart failure “Created with BioRender.com”.

Different types of NEP inhibitors are developed in the management of heart failure and hypertension and as analgesic agents.^11^ NEP inhibitors including sacubitril, RB‐101, omapatrilat, ecadotril, and candoxatril are rarely used alone but used in combination with AngII receptor blockers (ARBs) in the management of heart failure through modulation of RAS and expression of natriuretic peptides (Figure 2).

Sacubitril was the first NEP inhibitor approved in 2015 for the management of heart failure.^11^ Sacubitril in combination with valsartan was initially named LCZ696, which is an ARB and NEP inhibitor (ARNI). LCZ696 decreases blood pressure more than valsartan alone in hypertensive patients.^12^ In the PARADIGM‐HF study, LCZ696 was more effective than angiotensin‐converting enzyme inhibitor (ACEI) enalapril in the management of heart failure.^13^ The combo of NEP inhibitor/Valsartan (approved by the Food and Drug Administration in 2015) has been a significant addition to heart failure therapy in diabetes and no major complications have been seen.^13^

NEP inhibitors are also effective in the management of type 2 diabetes mellitus (T2DM) by increasing the circulating level of GLP‐1, which is degraded by NEP.^14^ However, the acute effect of NEP inhibitors leads to detrimental effects by increasing blood glucose independent of GLP‐1 increment.^15^ These findings suggest a controversial point regarding the potential role of NEP inhibitors in T2DM patients. Thus, this perspective aimed to elucidate the controversial points concerning the role of NEP inhibitors in T2DM.

## 2 ROLE OF PANCREATIC NEP IN T2DM

T2DM is a metabolic disorder characterized by hyperglycemia and insulin resistance (IR) due to dysfunction of pancreatic β cells and insulin insensitivity.^16^, ^17^ T2DM represents 90% of all diabetes and commonly occurs in adults aged >40 years.^18^ T2DM is associated with cardiometabolic disorders including obesity, hypertension, and dyslipidemia.^19^, ^20^ Accumulation of amyloids in the pancreatic β cells induces progressive pancreatic β cell loss with the development of pancreatic β cell dysfunction and overt T2DM.^21^ The chief constituent of pancreatic β cell amyloids is islet amyloid polypeptide (IAPP) also known as amylin, which is mainly cleaved by pancreatic NEP. Progressive deposition of IAPP triggers the development of pancreatic β cell toxicity.^21^ NEP blocks the deposition of extracellular pancreatic IAPP and prevents pancreatic β cell dysfunction. Therefore, NEP seems to be protective against the development of T2DM through modulation of pancreatic IAPP and accumulation of amyloids.^21^

Aggregation of IAPP in the pancreas leads to the development of T2DM whereas accumulation of Aβ in the brain promotes the development of AD.^22^, ^23^ Therefore, both T2DM and AD share a similarity in the pathogenesis of brain IR and cognitive dysfunction. In this state, AD is classified as type 3 diabetes mellitus.^22^, ^24^ IAPP is derived from pre‐pro‐IAPP to form 37 amino acid peptide.^25^ Notably, IAPP is co‐secreted with insulin from pancreatic β cells into circulation. Progressive accumulation of IAPP induces the formation of oligomers and fibrils with subsequent amyloid deposits observed in T2DM.^25^ Of interest, human IAPP lacks proline residues that prevent its deposition as seen in rat IAPP.^26^ Notoriously, impairment of human IAPP processing due to defects in the activity of autophagy and proteasome may contribute toward IAPP‐induced pancreatic β cytotoxicity.^25^ Therefore, restoration of IAPP proteostasis may be a promising option in the prevention and treatment of T2DM.^25^

**FIGURE 3:**
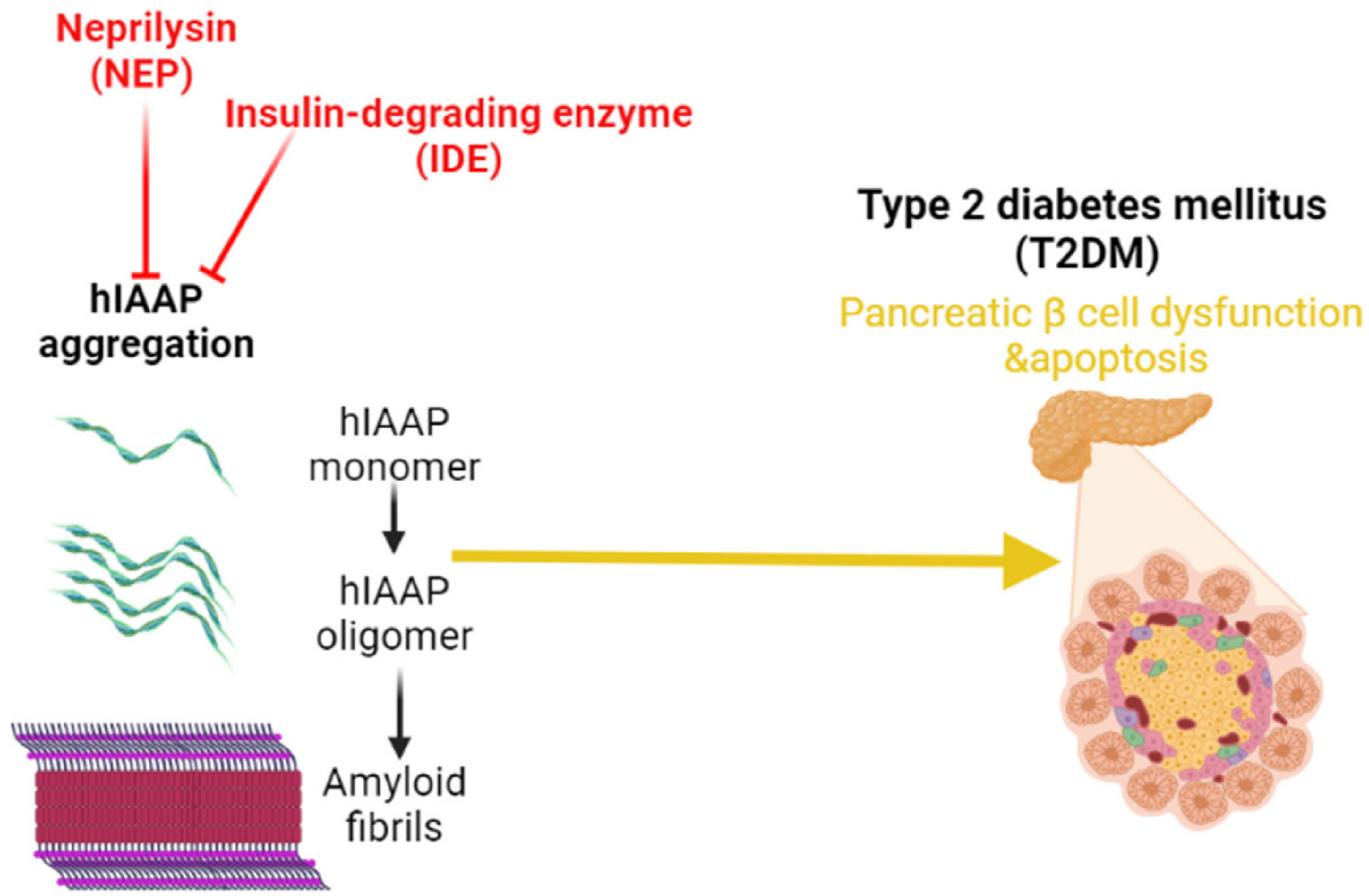
Human islet amyloid polypeptide (IAPP) and development of pancreatic β cell dysfunction “Created with BioRender.com”.

Degradation of IAPP is done by peptidase enzymes like an insulin‐degrading enzyme (IDE) and NEP to prevent the accumulation of IAPP in the pancreatic β cells and the development of T2DM.^21^ Downregulation or upregulation of these enzymes affects the functional capacity of IAPP. For example, inhibition of IDE by bacitracin accelerates amyloid deposition in insulinoma cell lines by inhibiting the degradation of pancreatic IAPP.^27^ NEP is highly expressed in the pancreatic β cells and is involved in the clearance of both pancreatic amyloids and brain Aβ (Figure 3).^3^, ^28^ Moreover, an experimental study with transgenic mice illustrated that inhibition of NEP promotes pancreatic β cell amyloidogenesis.^28^ However, NEP activators or upregulation of pancreatic β cell NEP reduce pancreatic β cell amyloidogenesis and apoptosis in cell lines.^28^ NEP prevents amyloid fibrilization in the pancreatic β cells by about 98% compared to neuronal Aβ fibrilization.^21^ NEP blocks human IAPP through noncatalytic and catalytic interactions.^21^ Intracellular accumulation of soluble IAPP in the pancreatic β cell is started initially.^29^ However, NEP inactivates extracellular IAPP, which is more intricate in the propagation of pancreatic β cytotoxicity.^30^ In vitro study demonstrated that IAPP was associated with β cell apoptosis through caspase‐3 activation. In addition, pancreatic β cells are more susceptible to IAPP toxicity than α cells cultured under the same conditions.^30^ As well, in vitro study conducted by Zraika et al^31^ observed that NEP inhibitors increase amyloid deposition in the pancreatic β cells by 54% and induce pancreatic β apoptosis by 75%. However, NEP activators or NEP upregulation led to a decrease in apoptosis and amyloid deposition in the pancreatic β cells by 79%. NEP reduces apoptosis and amyloid deposition in the pancreatic β cells through inhibition of IAPP fibril generation rather than degrading of IAPP.^31^ Recently, an experimental study conducted by Parilla et al^32^ revealed that NEP deficiency is linked with the expansion of pancreatic β cell mass in mice.

These findings suggest that NEP expression in the pancreatic β cells has a protective role against pancreatic β cell dysfunction and apoptosis through inhibition of pancreatic amyloidogenesis. Thus, NEP activators could be an effective therapeutic strategy in the prevention and treatment of T2DM.

## 3 NEP AND BLOOD GLUCOSE HOMEOSTASIS

It has been reported that NEP contributes to the impairment of glucose homeostasis through modulation of IR, pancreatic β cell mass, and development of pancreatic β cell dysfunction as observed in T2DM.^33^ Notably, the expression of NEP is increased in both T2DM and nutrient excess.^34^, ^35^ Different experiment studies revealed that NEP activity was augmented in mice fed on a high‐fat diet and correlated with the reduction of pancreatic β cell mass and development of IR.^34^, ^36^ NEP‐deficient mice experience good glycemic control with augmentation of GLP‐1 activity.^36^ Genetic ablation of NEP improves glucose homeostasis through the GLP‐1‐dependent pathway. As well, pharmacological inhibition of NEP in humans increases GLP‐1 activity.^37^ Therefore, inhibition of NEP may improve insulin sensitivity and glucose tolerance and protect pancreatic β cells from glucotoxicity.

Overexpression of NEP had been associated with the development of IR and metabolic syndrome in a study that involved 318 subjects with metabolic syndrome.^34^ NEP activity correlated with body mass index (BMI) and IR with increasing levels in subjects with multiple cardiovascular risk factors. NEP protein production in human adipocytes increased during cell differentiation and plasma. Thus, NEP is associated with cardiometabolic risk in the presence of IR and obesity.^34^ Therefore, NEP activity is positively correlated with IR and BMI in obese patients with cardiometabolic disorders.^23^, ^34^

Reduction of pancreatic β cell mass by the effect of NEP inhibitors is not fully elucidated; however, increasing NEP activity is associated with a compensatory increase in pancreatic β cell mass to overcome glucose dyshomeostasis.^38^ When overt T2DM is developed, the pancreatic β cell mass is reduced due to apoptosis.^38^ However, the experimental study confirmed that NEP deficiency or use of NEP inhibitors improves pancreatic β cell mass in mice through modulation expression of GLP‐1 receptors, which promotes survival, differentiation, and proliferation of pancreatic β cells.^32^ Therefore, NEP deficiency may increase pancreatic β cell mass by increasing the GLP‐1signaling pathway, which may explain the increase of pancreatic β cell mass in NEP deficient mice treated with a high‐fat diet.

**FIGURE 4:**
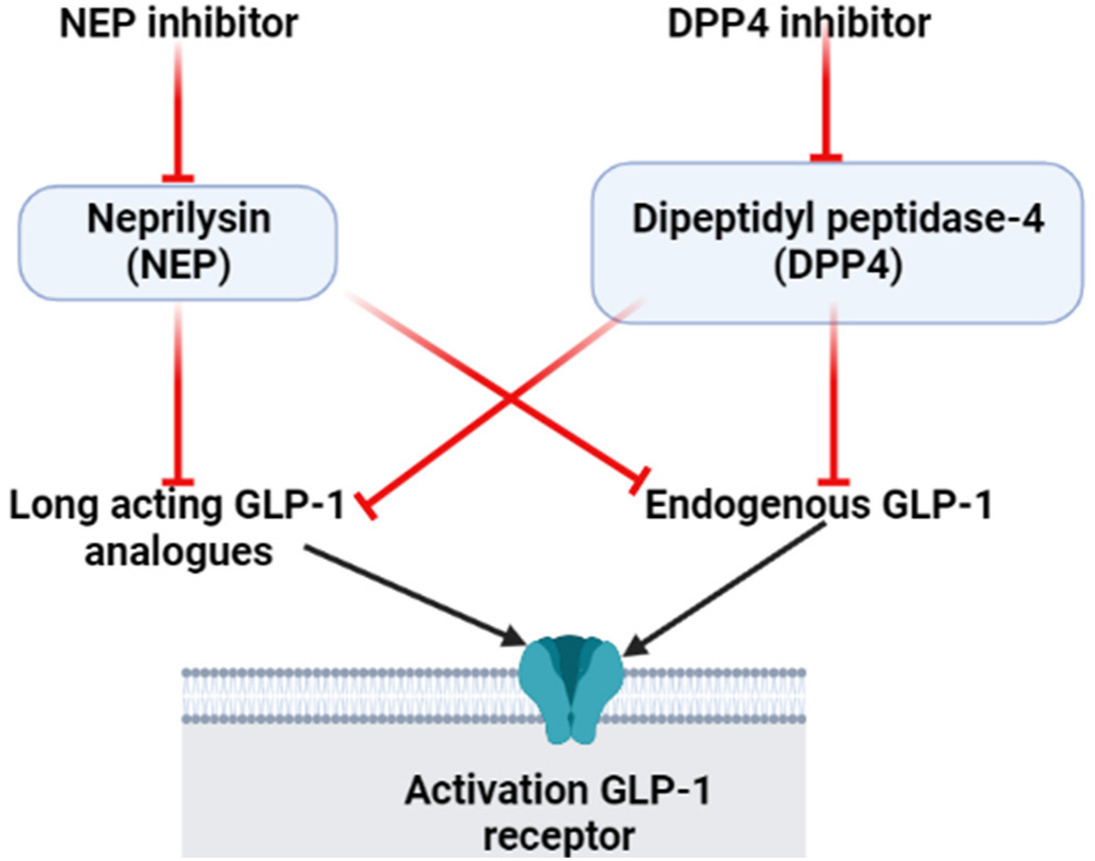
Role of neprilysin (NEP) and dipeptidyl peptidase‐4 (DPP4) in degrading of glucagon‐like peptide 1 (GLP‐1) “Created with BioRender.com”.

Furthermore, NEP increases dipeptidyl peptidase‐4 (DPP4) activity and contributes to increasing active GLP‐1 proteolysis.^36^ Therefore, the activity of plasma DPP4 is reduced in NEP‐deficient mice on a high‐fat diet.^36^ Thus, the acute effect of NEP inhibitors reduces DPP4 activity when NEP activity is exaggerated as in a high‐fat diet; however, under the normal physiological condition; there is no interaction between NEP and DPP4.^36^ Simonsen et al^39^ showed that NEP inhibitors did not improve glycemic control in diabetic rats on DPP4 inhibitors. Remarkably, 50% of GLP‐1 activity is reduced by the effect of NEP; thus the combination of NEP inhibitors with DPP4 inhibitors is more effective in maintaining GLP‐1 activity.^40^ An experimental study observed that NEP inhibitor candoxatril alone or in the combination with DPP4 inhibitor valine pyrrolidide increases GLP‐1. However, the combination was superior to the DPP4 inhibitor when used alone in the elevation of GLP‐1.^40^ These findings suggest that NEP inhibitors may improve glycemic control through increasing endogenous GLP‐1 activity and reduction of DPP4 activity (Figure 4 **)**.

In addition, NEP inhibitors increase other substrates cleaved by NEP‐like bradykinin, which improves insulin sensitivity in mice.^41^ Similarly, bradykinin through activation of the bradykinin 2 receptor enhances glucose uptake and oxidation.^42^ Wu et al^43^ demonstrated that bradykinin could protect endothelial cells from the effect of high blood glucose through modulation of the Akt pathway. Therefore, NEP inhibitors produce a protective effect against the development of diabetic complications via GLP‐1‐dependent and independent mechanisms.

However, different studies confirmed that NEP deficiency did not affect glycemic control and did not produce any benefit against high‐fat diet‐induced dysglycemia.^34^, ^44^ NEP‐deficient mice did not experience any changes in IR, glucose tolerance, and body/epidermal fat weight compared to the controls.^34^ Likewise, treatment with NEP inhibitors produces insignificant effects on glucose control in rats.^39^ Of interest, follow‐up of NEP‐deficient mice for 1 year showed they develop obesity and impairment of glucose tolerance.^45^

These findings indicated that NEP plays a minor role in the regulation of glucose homeostasis and insulin sensitivity. However, these experimental studies may not take into consideration the following points: pharmacological inhibition of NEP does not completely reduce NEP activity as in genetic ablation, and the duration of experimental studies regarding the use of NEP inhibitors and high diet period may affect the final data. These findings proposed that NEP inhibitors might not be effective in controlling blood glucose in T2DM patients.

## 4 NEP INHIBITORS AND GLUCOSE HOMEOSTASIS

### 4.1 The beneficial effects

It has been shown that NEP inhibitors can regulate blood glucose through modulation of pancreatic β cell function.^14^ Preclinical and clinical showed that NEP inhibitors alone or in combination with ARBs exerted beneficial effects on glucose tolerance and homeostasis^14^ (Table 1). As NEP inhibitors are approved for the management of heart failure, their use in the management of T2DM is regarded as a new therapeutic option.^14^ In vitro study illustrated that inhibition of NEP in isolated pancreatic β cells from mice promotes insulin secretion.^46^ As well, a high‐fat diet in NEP‐deficient mice results in better β cell function and insulin sensitivity.^32^ Of interest, the administration of intravenous NEP inhibitor racecadotril increases insulin secretion in a dose‐dependent manner in Wistar rats.^47^ As well, omapatrilat, a dual ACE and NEP inhibitor has been reported to show superior antihypertensive, antiatherosclerotic, insulin‐sensitizing, and cardiovascular and renoprotective effects to ACEIs in experimental animal models for diabetes.^48^ Therefore, NEP inhibitors may delay the progression of end‐organ damage in T2DM patients.

**TABLE 1:** Beneficial effects of NEP inhibitors.

Study type	Findings	Ref.
Review	NEP inhibitors alone or in combination with ARBs improve glucose homeostasis.	Esser et al^14^
In vitro study	Inhibition of NEP promotes insulin secretion.	Esser et al^46^
Experimental study	NEP‐deficient mice experience better insulin sensitivity.	Parilla et al^32^
Experimental study	NEP inhibitor increases insulin secretion in rats.	Wu et al^47^
Experimental study	Omapatrilat, a dual ACE and NEP, has superior insulin‐sensitizing effects compared to ACE inhibitors in experimental animals.	Malek et al^48^
Clinical trial studies	ARNIs had more cardioprotective and renoprotective effects compared to ARBs and ACEIs.	McMurray et al^49^; Packer et al^50^
Prospective study	T2DM patients with heart failure on ARNIs had a greater long‐term reduction in HbA_1c_ than those receiving enalapril.	Seferovic et al^51^
Prospective study	Long‐term therapy with ARNIs improves insulin sensitivity in obese hypertensive patients.	Jordan et al^52^
Prospective study	ARNIs use in T2DM patients for 3 months reduces NEP plasma levels.	Nougué et al^53^

Abbreviations: ACE, angiotensin‐converting enzyme; ARB, angiotensin receptor blocker; ARNI, angiotensin receptor/neprilysin inhibitor; HbA_1c_, glycated hemoglobin; NEP, neprilysin; T2DM. type 2 diabetes mellitus.

Clinical trial studies observed that ARNIs had cardioprotective and renoprotective effects compared to ARBs and ACEIs.^49^, ^50^ It has been shown that ARNIs improve glucose homeostasis, glucose control, and insulin sensitivity in T2DM patients.^14^ Furthermore, NEP inhibitors improve glucose tolerance through the modulation of insulin sensitivity and pancreatic β cell functions in humans.^14^ Of note, various human studies recommend the use of NEP inhibitors in the management of T2DM.^51^, ^52^ ARNIs use for 3 years produces helpful metabolic effects in T2DM patients as evidenced by the reduction of hemoglobin (HbA_1c_).^51^ PARADIGM‐HF (ENTRESTO) trial had 3778 diabetic patients with a decline of HbA_1c_ by 0.26%. There is extensive evidence from ACEI and ARB studies that similar HbA_1c_ reductions are seen with a variety of these agents, making it likely that valsartan alone is responsible.^51^ However, T2DM patients with heart failure enrolled in PARADIGM‐HF who received ARNIs had a greater long‐term reduction in HbA_1c_ than those receiving enalapril.^51^ These findings suggest that ARNIs might enhance glycemic control in patients with diabetes and heart failure. Similarly, long‐term therapy with ARNIs in obese hypertensive patients improves lipid profile and insulin sensitivity.^52^ A prospective study involved 73 chronic heart failure patients who were switched from ACEIs or ARBs to ARNIs confirmed that ARNIs treatment leads to a metabolic benefit in the study population and supports the relevance of NEP inhibition along with AT_1_‐receptor blockade in the regulation of human glucose and lipid metabolism.^52^ A clinical study involving 73 patients with heart failure including 16 T2DM patients treated with ARNIs for 3 months showed reduced NEP plasma level and biomarker of protein glycation.^53^ Moreover, NEP inhibitors exerted protective effects against T2DM‐related complications like vasculopathy, nephropathy, neuropathy, and cardiomyopathy.^48^ The findings proposed that ARNIs are superior to ACEIs or ARBs in patients with heart failure by reducing IR and increasing cardioprotective substrates degraded by NEP. However, the precise mechanistic effects of NEP inhibitors on glucose homeostasis are not fully elucidated.

#### 4.1.1 The possible mechanisms

The potential mechanism related to the effect of NEP inhibitors on glucose homeostasis is linked to the increase of many substrates that are normally degraded by NEP (Table 2). These substrates like bradykinin, GLP‐1, and natriuretic peptides (NPs) may increase insulin sensitivity and pancreatic β cell function.^14^ Augmentation of GLP‐1 by NEP inhibitors is the most important mechanism for controlling glucose homeostasis.^54^ As well, GLP‐1 is high in NEP‐deficient mice suggesting that pancreatic NEP plays a critical role in the reduction of gut‐derived GLP‐1.^54^ In addition, DPP4 activity is also activated by pancreatic NEP^36^; thus NEP inhibitors may increase GLP‐1 and decrease DPP4 activity leading to improvement of insulin sensitivity and pancreatic β cell functions in humans as supported by recent studies showed that ARNI was effective in T2DM patients.^14^ Under conditions of increased dietary fat, an improved glycemic status in NEP‐deficient mice was linked with elevated active GLP‐1 levels, reduced plasma DPP‐4 activity, and improved β cell function. Therefore, NEP inhibitors may be a novel management strategy for T2DM.^36^

**TABLE 2:** Possible mechanisms of beneficial effects of NEP inhibitors.

Study type	Findings	Ref.
Experimental study	NEP inhibitors increase GLP‐1 level.	Hupe‐Sodmann et al^54^
Experimental study	NEP inhibitors insulin sensitivity by increasing of GLP‐1 and decrease DPP4 activity.	Willard et al^36^
Experimental study	Dual inhibition of DPP4 and NEP increases the half‐life of GLP‐1.	Plamboeck et al^40^
A randomized, controlled study	Augmentation of GIP by NEP inhibitors improves glucose homeostasis and insulin sensitivity in T2DM patients.	Frias et al^56^
Review	NEP inhibitors improve glucose homeostasis and insulin sensitivity by increasing level of NPs.	Moro et al^60^
Review	NEP inhibitors improve insulin sensitivity and glucose homeostasis by increasing bradykinin level.	Campbell^64^
Review	LCZ696 can enhance insulin sensitivity and glucose homeostasis by increasing oxytocin serum level.	Dargad et al^68^

Abbreviations: DPP4, dipeptidyl peptidase‐4; GIP, glucose‐dependent insulinotropic peptide; GLP‐1, glucagon‐like peptide 1; NEP, neprilysin; NP, natriuretic peptide; T2DM, type 2 diabetes mellitus.

To increase the efficacy of NEP inhibitors in the management of T2DM, they may be combined with antidiabetic agents like metformin and DPP4 inhibitors. Of note, NEP inhibitors plus DPP4 inhibitors may lead to an additive effect to achieve more glucose control in T2DM patients through augmentation of GLP‐1.^55^ DPP4 cleaves only active GLP‐1 whereas NEP cleaves both active and truncated GLP‐1. Thus, dual inhibition of DPP4 and NEP increases the half‐life of GLP‐1 more compared to NEP inhibitors alone.^40^

Glucose‐dependent insulinotropic peptide (GIP) and pancreatic polypeptide (PP) are also increased by NEP inhibitors.^54^ Augmentation of GIP may improve glucose homeostasis and insulin sensitivity.^56^ A randomized double‐blind controlled trial for use of tirzepatide, a dual GIP and GLP‐1 agonist, for 12 weeks in T2DM patients showed that this drug improves insulin sensitivity and atherogenic profile.^56^ However, impairment of glucose tolerance is correlated with enhanced postprandial PP^57^ suggesting a compensatory mechanism to overcome glucose intolerance and hyperglycemia. Different studies revealed that injury of PP cells in chronic pancreatitis correlates with pancreatic β cell dysfunction and the development of overt T2DM.^58^, ^59^ Therefore, NEP inhibitors may modulate GIP and PP in response to blood glucose and metabolic disturbance in T2DM patients.

Furthermore, the reduction of NPs is linked with the development of T2DM as these peptides are involved in the regulation of metabolic and cardiovascular effects.^60^ NPs are reduced in obesity and regarded as a risk predictive factor for the development of T2DM. NPs improve glucose homeostasis and insulin sensitivity; thus increasing circulating NPs levels by NEP inhibitors could be effective in the management of T2DM. NEP is the major enzymatic pathway for the degradation of NPs.^61^ The NPs act as endogenous inhibitors of the RAS; thus inhibition of NEP increases levels of NPs.^61^ Through simultaneously inhibiting the RAS and potentiating the NPs, NEP inhibitors reduce vasoconstriction, enhance vasodilation, improve sodium/water balance, and, in turn, decrease peripheral vascular resistance and blood pressure and improve local blood flow.^61^ Thus, the combined inhibition of ACE and NEP is a new and promising approach to treat patients with hypertension, atherosclerosis, or heart failure. Besides, reduced plasma NPs level is observed in metabolic diseases such as obesity and T2DM.^62^ A low circulating NPs level also predicts the risk of new‐onset T2DM. NPs can activate a thermogenic program in brown and white fat, increase energy expenditure, inhibit food intake, and regulate glucose homeostasis.^62^ In addition, NPs improve blood glucose control and insulin sensitivity by increasing glucose uptake in human adipocytes.^63^ Therefore, targeting of the NP system could be a novel avenue for the management of obesity and T2DM.^60^

Similarly, bradykinin is also increased following the use of NEP inhibitors^14^ this increment in bradykinin is associated with the improvement of glucose homeostasis and insulin sensitivity.^42^ Bradykinin reduces peripheral IR through the improvement of peripheral glucose uptake and oxidation.^42^ It has been shown that LCZ696 produces angioedema caused by increasing bradykinin levels with an incidence at least equal to that of ACEI therapy.^64^ However, LCZ696‐induced increment in bradykinin could be beneficial in controlling blood glucose and insulin sensitivity.^64^ Thus, NEP inhibitors may improve insulin sensitivity and glucose homeostasis through the bradykinin‐dependent pathway.

In addition, oxytocin is highly degraded by NEP^65^; therefore increasing circulating oxytocin by NEP inhibitors is expected. Of note, oxytocin has a cardioprotective effect on T2DM and obesity.^66^ Oxytocin activates peripheral glucose uptake and reduces IR in mice.^66^ Al‐kuraishy et al^67^ illustrated that oxytocin was superior to GLP‐1 in the management of T2DM in COVID‐19 patients. As well, oxytocin has anti‐inflammatory and antioxidant effects, thereby attenuating T2DM‐mediated complications.^67^ A review study suggested that LCZ696 can enhance insulin sensitivity and glucose homeostasis by increasing oxytocin serum levels.^68^ In this state, augmentation of oxytocin levels by NEP inhibitors could be a new therapeutic strategy in the management of T2DM.

Taken together, NEP inhibitors have beneficial effects on glucose homeostasis and insulin sensitivity by increasing various substrates including GLP‐1 and thus, could be effective alone or in combination with antidiabetic agents in treating T2DM patients.

### 4.2 The detrimental effects

NEP inhibitors may be not effective or produce a neutral effect on glucose homeostasis and insulin sensitivity (Table 3).^45^ NEP‐deficient mice start to become obese under a normocaloric diet at the age of 6–7 months.^45^ NEP‐deficient mice are susceptible to the development of late‐onset obesity and impairment of glucose tolerance.^69^ Simonsen et al^39^ observed that NEP inhibitors did not improve glycemic indices compared to DPP4 inhibitors in rats. Similarly, intraperitoneal administration of NEP inhibitors in rats with T2DM or on a high‐fat diet did not produce beneficial effects on glucose homeostasis and insulin sensitivity.^70^ The underlying explanation for the poor effect of NEP inhibitors in these experimental studies might be related to reducing the efficacy of NEP inhibitors by these routes or to the exaggeration of compensatory mechanisms against the use of NEP inhibitors that need time to subside. As well, the use of intraperitoneal routes in these experimental studies may reduce the stimulatory effect of NEP inhibitors on gut‐derived peptides. Thus, the ultimate conclusion regarding the effect of NEP inhibitors on glucose homeostasis and insulin sensitivity needs to be reevaluated. Recently, Albrechtsen et al^15^ observed that acute administration of ARNIs in T2DM patients may not depend on the alterations of enteropancreatic hormones, and NEP inhibitors may lead to hyperglycemia due to an increment in glucagon serum levels. A crossover trial involving 12 T2DM patients with obesity with different interventions including ARNIs alone or ARNIs plus DPP4 inhibitors showed that ARNIs increase postprandial blood glucose by 57% with increasing levels of glucagon, C‐peptide, and GLP‐1 without effect on GIP and insulin levels.^15^ Furthermore, acute NEP inhibition and ARB treatment worsen rather than improve postprandial glucose control in T2DM patients.^71^ Likewise, long‐term ARNIs treatment in T2DM patients with heart failure may improve glycemic control by specific mechanisms that vary from those provoked during acute administration. In contrast, acute inhibition of NEP in T2DM patients results in hyperglycemia independent of GLP‐1 level.^71^

**TABLE 3:** Detrimental effects of neprilysin (NEP) inhibitors.

Study type	Findings	Ref.
Experimental study	NEP inhibitors deteriorate glucose homeostasis and insulin sensitivity.	Becker et al^45^
Experimental study	NEP‐deficient mice are susceptible to the development of obesity and impairment of glucose tolerance.	Poorgolizadeh et al^69^
Experimental study	NEP inhibitors did not improve glycemic indices in rats.	Simonsen et al^39^
Experimental study	NEP inhibitors did not improve glucose homeostasis and insulin sensitivity in rats with T2DM.	Davidson et al^70^
A prospective study	Administration of ARNIs lead to hyperglycemia in T2DM patients.	Albrechtsen et al^15^
A prospective, crossover study	ARNIs worsen postprandial glucose control in T2DM patients.	Wewer et al^71^

Abbreviations: ARNI, angiotensin receptor/neprilysin inhibitor; NEP, neprilysin; T2DM. type 2 diabetes mellitus.

#### 4.2.1 The possible mechanisms

The underlying mechanism for the detrimental effects of NEP inhibitors is due to alterations of some substrates degraded by NEP that may affect glucose homeostasis and insulin sensitivity (Table 4). For example, NEP is necessary for the conversion of vasoconstrictor and proinflammatory AngII to vasodilator and anti‐inflammatory Ang1‐7.^72^, ^73^ In addition, NEP promotes the conversion of Ang1‐7 to Ang3‐4, Ang1‐4, and Ang 5–7. Ang1‐7 and their metabolites improve pancreatic β cell functions and enhance glucose‐stimulated insulin secretion.^72^, ^74^ However, administration of Ang1‐7 in NEP‐deficient mice failed to promote glucose‐stimulated insulin secretion, and NEP‐derived Ang1‐4 did not produce glucose‐stimulated insulin secretion in NEP‐deficient mice.^72^ This finding suggests that Ang1‐7 and Ang1‐4 are further cleaved by NEP to Ang1‐2, which has an insulinotropic effect.^75^ Besides, NEP inhibitors increase AngII levels, which inhibits pancreatic β cell functions and promotes the development of IR.^74^, ^76^ Therefore, NEP is necessary for Ang1‐7‐dependent insulin secretion. Thus, NEP inhibitors may affect glucose homeostasis through the inhibition of the generation of Ang1‐7.

**TABLE 4:** Possible mechanisms of detrimental effects of NEP inhibitors.

Study type	Findings	Ref.
Experimental study	NEP inhibitors reduced Ang1‐2 which has an insulinotropic effect.	Shao et al^75^
Experimental study	NEP inhibitors increase AngII levels, which inhibits pancreatic β cell functions.	He et al^76^
In vitro and experimental studies	NEP inhibitors have a harmful effect on glucose tolerance by increasing adrenomedullin expression.	Aggarwal et al^77^
A prospective study	NEP inhibitors increase the level of glucagon, which impairs glucose tolerance.	Albrechtsen et al^15^
Experimental study	NEP inhibitors increase amyloid deposition in pancreatic β cells.	Parilla et al^32^

Abbreviations: AngII, angiotensin II; NEP, neprilysin.

In addition, adrenomedullin, which is increased by NEP inhibitors, has a harmful effect on glucose tolerance through the inhibition of insulin secretion.^77^ Adrenomedullin is a peptide hormone highly expressed in vasculature involved in the regulation of inflammatory response, insulin release and glucose metabolism.^78^ Normally, adrenomedullin expression is low in healthy subjects and elevated in T2DM patients and associated complications.

Similarly, NEP inhibitors increase the level of glucagon, which is normally degraded by NEP.^79^ Interestingly, NEP inhibitors increase postprandial amino acids catabolism without effect on glucose homeostasis.^79^ However, the acute effect of NEP inhibitors leads to postprandial hyperglycemia by increasing glucagon levels.^15^ Thus, hyperglucagonemia may reduce the beneficial effect of NEP inhibitors mediated by increasing GLP‐1.^80^

Moreover, NEP inhibitors increase amyloid deposition in pancreatic β by 54% and induce apoptosis of pancreatic β cells by 75%. However, NEP activators or NEP upregulation led to a decreased apoptosis and amyloid deposition in the pancreatic β by 79%.^31^ NEP decreases apoptosis and amyloid deposition in the pancreatic β through inhibition of IAPP fibril generation rather than degrading of IAPP.^31^ Parilla et al^32^ revealed that NEP deficiency is linked with the expansion of pancreatic β cell mass in mice. Therefore, prolonged use of NEP inhibitors can increase the risk of T2DM development via amyloid deposition and induction of pancreatic β dysfunction.

## 5 PROS AND CONS OF NEP INHIBITORS' EFFECTS ON GLUCOSE HOMEOSTASIS

Regarding the safety profile of NEP inhibitors, it has been shown that long‐term use of NEP inhibitors may increase IAPP aggregation with the development of pancreatic β cell dysfunction and apoptosis.^31^ However, NEP inhibitor‐induced GLP‐1 elevation may reduce pancreatic β cell apoptosis.^81^ These findings from experimental studies are not confirmed in human studies. Furthermore, NEP inhibitors affect the clearance of brain Aβ,^69^ which raises a concern about the risk of AD in relation to long‐term therapy with NEP inhibitors. Therefore, long‐term follow‐up for the effects of NEP inhibitors on cognitive function and AD risk is recommended. Long‐term and short‐term effects of NEP inhibitors may lead to detrimental effect on insulin sensitivity and glucose homeostasis through different mechanisms including augmentation of substrates that adversely affect insulin signaling and glucose homeostasis. Furthermore, long‐term use of NEP inhibitors may increase the risk for the development of T2DM by increasing the deposition of pancreatic IAPP. To reduce the detrimental effects of NEP inhibitors and increase their efficacy, NEP inhibitors might be combined with ARBs. This combination reduces the harmful adverse effects mediated by increasing AngII levels.^53^ In addition, ARNIs are more effective than NEP inhibitors on insulin sensitivity and glucose homeostasis.^82^

Taken together, NEP inhibitors can affect insulin sensitivity and glucose homeostasis in bidirectional ways that could be beneficial or harmful depending on the time, duration, dose, and route of administration. Therefore, a large‐scale prospective study to elucidate the effects of NEP inhibitors or ARNIs on glucose homeostasis in both diabetic patients and healthy subjects is recommended. As well, long‐term follow‐up of patients receiving NEP inhibitors or ARNIs by measuring IAPP serum level is advisable to outweigh the risk/benefit ratio in relation to AD risk.

## 6 CONCLUSIONS

NEP enzyme inactivates various peptide hormones including NPs, bradykinin, oxytocin, neurotensin, substance P, enkephalins, AngII, ET‐1, and GLP‐1. NEP expression in the pancreatic β cells has a protective role against pancreatic β cell dysfunction and apoptosis through inhibition of pancreatic amyloidogenesis. Overexpression of NEP enzyme is associated with development of IR, poor glucose homeostasis, and cardiometabolic disorders by the increase DPP4 activity and reduction of circulating GLP‐1. Therefore, NEP inhibitors improve glucose homeostasis by inhibition of DPP4 activity and increasing of circulating GLP‐1. NEP inhibitors are rarely used alone but are used in combination with ARBs in the management of heart failure through modulation of RAS and expression of natriuretic peptides. Taken together, NEP inhibitors produce beneficial rather than detrimental effects on glucose homeostasis. Therefore, large prospective studies are suggested in this regard.

## AUTHOR CONTRIBUTIONS

Hayder M. Al‐kuraishy and Ali I. Al‐Gareeb: conceptualization and writing of the original draft. Athanasios Alexiou, Marios Papadakis, Hebatallah M. Saad, and Gaber El‐Saber Batiha: preparation of figures; writing, correcting, and amending of the article. Faisal Holil AlAnazi, Hanan A. Ogaly, and Yousef Abud Alanazi: editing and polishing of the manuscript and responding to reviewers' comments. All authors contributed significantly to the manuscript and approved the submitted version.

## FUNDING INFORMATION

Open Access funding enabled and organized by Projekt DEAL. This work was supported by the University of Witten‐Herdecke Germany.

## References

[1] Nalivaeva N , Zhuravin I , Turner A . Neprilysin expression and functions in development, ageing and disease. Mech Ageing Dev. 2020;192:111363. 32987038 10.1016/j.mad.2020.111363 PMC7519013

[2] Wang Y , Zhou R , Lu C , Chen Q , Xu T , Li D . Effects of the angiotensin‐receptor neprilysin inhibitor on cardiac reverse remodeling: meta‐analysis. J Am Heart Assoc. 2019;8(13):e012272. 31240976 10.1161/JAHA.119.012272 PMC6662364

[3] Alsubaie N , Al‐kuraishy HM , Al‐Gareeb AI , et al. Statins use in Alzheimer disease: bane or boon from frantic search and narrative review. Brain Sci. 2022;12(10):1290. 36291224 10.3390/brainsci12101290 PMC9599431

[4] Solomon SD , McMurray JJ , Anand IS , et al. Angiotensin–neprilysin inhibition in heart failure with preserved ejection fraction. N Engl J Med. 2019;381(17):1609‐1620. 31475794 10.1056/NEJMoa1908655

[5] Pardossi‐Piquard R , Dunys J , Yu G , St. George‐Hyslop P , Alves da Costa C , Checler F . Neprilysin activity and expression are controlled by nicastrin. J Neurochem. 2006;97(4):1052‐1056. 16606360 10.1111/j.1471-4159.2006.03822.x

[6] Bayes‐Genis A , Prickett TC , Richards AM , Barallat J , Lupón J . Soluble neprilysin retains catalytic activity in heart failure. J Heart Lung Transplant. 2016;35(5):684‐685. 26830756 10.1016/j.healun.2015.12.015

[7] Alomair BM , Al‐kuraishy HM , Al‐Gareeb AI , et al. Montelukast and acute coronary syndrome: the endowed drug. Pharmaceuticals. 2022;15(9):1147. 36145367 10.3390/ph15091147 PMC9500901

[8] Bayés‐Genís A , Barallat J , Galán A , et al. Soluble neprilysin is predictive of cardiovascular death and heart failure hospitalization in heart failure patients. J Am Coll Cardiol. 2015;65(7):657‐665. 25677426 10.1016/j.jacc.2014.11.048

[9] Goliasch G , Pavo N , Zotter‐Tufaro C , et al. Soluble neprilysin does not correlate with outcome in heart failure with preserved ejection fraction. Eur J Heart Fail. 2016;18(1):89‐93. 26725876 10.1002/ejhf.435

[10] Vodovar N , Seronde M‐F , Laribi S , et al. Elevated plasma B‐type natriuretic peptide concentrations directly inhibit circulating neprilysin activity in heart failure. *JACC* . Heart Failure. 2015;3(8):629‐636. 26251090 10.1016/j.jchf.2015.03.011

[11] Revuelta‐López E , Núñez J , Gastelurrutia P , et al. Neprilysin inhibition, endorphin dynamics, and early symptomatic improvement in heart failure: a pilot study. ESC Heart Failure. 2020;7(2):559‐566. 32045114 10.1002/ehf2.12607 PMC7160502

[12] Campbell DJ . Long‐term neprilysin inhibition—implications for ARNIs. Nat Rev Cardiol. 2017;14(3):171‐186. 27974807 10.1038/nrcardio.2016.200

[13] Simpson J , Jhund PS , Silva Cardoso J , et al. Comparing LCZ696 with enalapril according to baseline risk using the MAGGIC and EMPHASIS‐HF risk scores: an analysis of mortality and morbidity in PARADIGM‐HF. J Am Coll Cardiol. 2015;66(19):2059‐2071. 26541915 10.1016/j.jacc.2015.08.878

[14] Esser N , Zraika S . Neprilysin inhibition: a new therapeutic option for type 2 diabetes? Diabetologia. 2019;62(7):1113‐1122. 31089754 10.1007/s00125-019-4889-y PMC6579747

[15] Albrechtsen NJW , Møller A , Martinussen C , et al. Acute effects on glucose tolerance by neprilysin inhibition in patients with type 2 diabetes. Diabetes Obes Metab. 2020;24(10):2017‐2026. 10.1111/dom.14789 PMC9545540 35676803

[16] Al‐Kuraishy HM , Al‐Gareeb AI , Alblihed M , Guerreiro SG , Cruz‐Martins N , Batiha GE‐S . COVID‐19 in relation to hyperglycemia and diabetes mellitus. Front Cardiovasc Med. 2021;8:644095. 34124187 10.3389/fcvm.2021.644095 PMC8189260

[17] Batiha GE‐S , Al‐kuraishy HM , Al‐Maiahy TJ , et al. Plasminogen activator inhibitor 1 and gestational diabetes: the causal relationship. Diabetol Metab Syndr. 2022;14(1):127. doi:10.1186/s13098-022-00900-2 36076264 PMC9454110

[18] Al‐Kuraishy HM , Hussian NR , Al‐Naimi MS , Al‐Gareeb AI , Al‐Mamorri F , Al‐Buhadily AK . The potential role of pancreatic γ‐aminobutyric acid (GABA) in diabetes mellitus: a critical reappraisal. Int J Prev Med. 2021;12:19. 34084316 10.4103/ijpvm.IJPVM_278_19 PMC8106282

[19] Al‐Kuraishy HM , Sami OM , Hussain NR , Al‐Gareeb AI . Metformin and/or vildagliptin mitigate type II diabetes mellitus induced‐oxidative stress: the intriguing effect. J Adv Pharm Technol Res. 2020;11(3):142‐147. 33102198 10.4103/japtr.JAPTR_18_20 PMC7574736

[20] Hussien NR , Al‐Naimi MS , Rasheed HA , Al‐Kuraishy HM , Al‐Gareeb AI . Sulfonylurea and neuroprotection: the bright side of the moon. J Adv Pharm Technol Res. 2018;9(4):120‐123. 30637228 10.4103/japtr.JAPTR_317_18 PMC6302683

[21] Guan H , Chow K , Shah R , Rhodes C , Hersh L . Degradation of islet amyloid polypeptide by neprilysin. Diabetologia. 2012;55(11):2989‐2998. 22898766 10.1007/s00125-012-2678-y PMC3660010

[22] Nguyen TT , Ta QTH , Nguyen TKO , Nguyen TTD , Van Giau V . Type 3 diabetes and its role implications in Alzheimer's disease. Int J Mol Sci. 2020;21(9):3165. 32365816 10.3390/ijms21093165 PMC7246646

[23] Al‐Kuraishy HM , Al‐Gareeb AI , Alsayegh AA , et al. A potential link between visceral obesity and risk of Alzheimer's disease. Neurochem Res. 2022;48:745‐766. doi:10.1007/s11064-022-03817-4 36409447

[24] Al‐kuraishy HM , Al‐Gareeb AI , Saad HM , Batiha GE‐S . Benzodiazepines in Alzheimer's disease: beneficial or detrimental effects. Inflammopharmacology. 2022;31:221‐230. doi:10.1007/s10787-022-01099-4 36418599

[25] Milardi D , Gazit E , Radford SE , et al. Proteostasis of islet amyloid polypeptide: a molecular perspective of risk factors and protective strategies for type II diabetes. Chem Rev. 2021;121(3):1845‐1893. 33427465 10.1021/acs.chemrev.0c00981 PMC10317076

[26] Westermark P , Engström U , Johnson KH , Westermark GT , Betsholtz C . Islet amyloid polypeptide: pinpointing amino acid residues linked to amyloid fibril formation. Proc Natl Acad Sci. 1990;87(13):5036‐5040. 2195544 10.1073/pnas.87.13.5036 PMC54256

[27] Bennett RG , Hamel FG , Duckworth WC . An insulin‐degrading enzyme inhibitor decreases amylin degradation, increases amylin‐induced cytotoxicity, and increases amyloid formation in insulinoma cell cultures. Diabetes. 2003;52(9):2315‐2320. 12941771 10.2337/diabetes.52.9.2315

[28] Zraika S , Hull RL , Udayasankar J , et al. Identification of the amyloid‐degrading enzyme neprilysin in mouse islets and potential role in islet amyloidogenesis. Diabetes. 2007;56(2):304‐310. 17259373 10.2337/db06-0430

[29] Westermark P , Andersson A , Westermark GT . Islet amyloid polypeptide, islet amyloid, and diabetes mellitus. Physiol Rev. 2011;91(3):795‐826. 21742788 10.1152/physrev.00042.2009

[30] Law E , Lu S , Kieffer T , et al. Differences between amyloid toxicity in alpha and beta cells in human and mouse islets and the role of caspase‐3. Diabetologia. 2010;53(7):1415‐1427. 20369225 10.1007/s00125-010-1717-9

[31] Zraika S , Aston‐Mourney K , Marek P , et al. Neprilysin impedes islet amyloid formation by inhibition of fibril formation rather than peptide degradation. J Biol Chem. 2010;285(24):18177‐18183. 20400513 10.1074/jbc.M109.082032 PMC2881741

[32] Parilla JH , Hull RL , Zraika S . Neprilysin deficiency is associated with expansion of islet β‐cell mass in high fat‐fed mice. J Histochem Cytochem. 2018;66(7):523‐530. 29553871 10.1369/0022155418765164 PMC6055259

[33] Kahn S . The relative contributions of insulin resistance and beta‐cell dysfunction to the pathophysiology of type 2 diabetes. Diabetologia. 2003;46(1):3‐19. 12637977 10.1007/s00125-002-1009-0

[34] Standeven KF , Hess K , Carter AM , et al. Neprilysin, obesity and the metabolic syndrome. Int J Obes (Lond). 2011;35(8):1031‐1040. 21042321 10.1038/ijo.2010.227 PMC3040694

[35] Antezana MA , Sullivan SR , Usui ML , et al. Neutral endopeptidase activity is increased in the skin of subjects with diabetic ulcers. J Invest Dermatol. 2002;119(6):1400‐1404. 12485446 10.1046/j.1523-1747.2002.19618.x

[36] Willard JR , Barrow BM , Zraika S . Improved glycaemia in high‐fat‐fed neprilysin‐deficient mice is associated with reduced DPP‐4 activity and increased active GLP‐1 levels. Diabetologia. 2017;60(4):701‐708. 27933334 10.1007/s00125-016-4172-4 PMC5342915

[37] Vodovar N , Nougue H , Launay J‐M , Solal AC , Logeart D . Sacubitril/valsartan in PARADIGM‐HF. Lancet Diabetes Endocrinol. 2017;5(7):495‐496. 10.1016/S2213-8587(17)30177-8 28645438

[38] Jurgens CA , Toukatly MN , Fligner CL , et al. β‐Cell loss and β‐cell apoptosis in human type 2 diabetes are related to islet amyloid deposition. Am J Pathol. 2011;178(6):2632‐2640. 21641386 10.1016/j.ajpath.2011.02.036 PMC3123989

[39] Simonsen L , Pilgaard S , Carr R , Kanstrup A , Holst J , Deacon C . Inhibition of neutral endopeptidase 24.11 does not potentiate the improvement in glycemic control obtained with dipeptidyl peptidase‐4 inhibition in diabetic Goto–Kakizaki rats. Horm Metab Res. 2009;41(11):851‐853. 19579179 10.1055/s-0029-1225609

[40] Plamboeck A , Holst J , Carr R , Deacon C . Neutral endopeptidase 24.11 and dipeptidyl peptidase IV are both mediators of the degradation of glucagon‐like peptide 1 in the anaesthetised pig. Diabetologia. 2005;48(9):1882‐1890. 16025254 10.1007/s00125-005-1847-7

[41] Arbin V , Claperon N , Fournié‐Zaluski M‐C , Roques BP , Peyroux J . Effects of dual angiotensin‐converting enzyme and neutral endopeptidase 24‐11 chronic inhibition by mixanpril on insulin sensitivity in lean and obese Zucker rats. J Cardiovasc Pharmacol. 2003;41(2):254‐264. 12548087 10.1097/00005344-200302000-00015

[42] Gregnani MF , Hungaro TG , Martins‐Silva L , Bader M , Araujo RC . Bradykinin B2 receptor signaling increases glucose uptake and oxidation: evidence and open questions. Front Pharmacol. 2020;11:1162. 32848770 10.3389/fphar.2020.01162 PMC7417865

[43] Wu Y , Fu C , Li B , et al. Bradykinin protects human endothelial progenitor cells from high‐glucose‐induced senescence through B2 receptor‐mediated activation of the Akt/eNOS signalling pathway. J Diabetes Res. 2021;2021:1‐13. 10.1155/2021/6626627 PMC8452971 34557552

[44] Davidson E , Coppey L , Lu B , et al. The roles of streptozotocin neurotoxicity and neutral endopeptidase in murine experimental diabetic neuropathy. Exp Diabetes Res. 2009;2009:1‐9. 10.1155/2009/431980 PMC2817866 20148083

[45] Becker M , Siems W‐E , Kluge R , et al. New function for an old enzyme: NEP deficient mice develop late‐onset obesity. PLoS One. 2010;5(9):e12793. 20862277 10.1371/journal.pone.0012793 PMC2940827

[46] Esser N , Barrow BM , Choung E , Shen NJ , Zraika S . Neprilysin inhibition in mouse islets enhances insulin secretion in a GLP‐1 receptor dependent manner. Islets. 2018;10(5):175‐180. 30142012 10.1080/19382014.2018.1502521 PMC6284476

[47] Wu H , Chang C , Cheng K , Yeh C , Cheng J . Increase of plasma insulin by racecadotril, an inhibitor of enkephalinase, in wistar rats. Horm Metab Res. 2010;42(04):261‐267. 20143290 10.1055/s-0029-1246190

[48] Malek V , Gaikwad AB . Neprilysin inhibitors: a new hope to halt the diabetic cardiovascular and renal complications? Biomed Pharmacother. 2017;90:752‐759. 28419972 10.1016/j.biopha.2017.04.024

[49] McMurray JJ , Packer M , Desai AS , et al. Angiotensin–neprilysin inhibition versus enalapril in heart failure. N Engl J Med. 2014;371:993‐1004. 25176015 10.1056/NEJMoa1409077

[50] Packer M , Claggett B , Lefkowitz MP , et al. Effect of neprilysin inhibition on renal function in patients with type 2 diabetes and chronic heart failure who are receiving target doses of inhibitors of the renin‐angiotensin system: a secondary analysis of the PARADIGM‐HF trial. Lancet Diabetes Endocrinol. 2018;6(7):547‐554. 29661699 10.1016/S2213-8587(18)30100-1

[51] Seferovic JP , Claggett B , Seidelmann SB , et al. Effect of sacubitril/valsartan versus enalapril on glycaemic control in patients with heart failure and diabetes: a post‐hoc analysis from the PARADIGM‐HF trial. Lancet Diabetes Endocrinol. 2017;5(5):333‐340. 28330649 10.1016/S2213-8587(17)30087-6 PMC5534167

[52] Jordan J , Stinkens R , Jax T , et al. Improved insulin sensitivity with angiotensin receptor neprilysin inhibition in individuals with obesity and hypertension. Clin Pharmacol Ther. 2017;101(2):254‐263. 27542885 10.1002/cpt.455

[53] Nougué H , Pezel T , Picard F , et al. Effects of sacubitril/valsartan on neprilysin targets and the metabolism of natriuretic peptides in chronic heart failure: a mechanistic clinical study. Eur J Heart Fail. 2019;21(5):598‐605. 30520545 10.1002/ejhf.1342

[54] Hupe‐Sodmann K , McGregor GP , Bridenbaugh R , et al. Characterisation of the processing by human neutral endopeptidase 24.11 of GLP‐1 (7–36) amide and comparison of the substrate specificity of the enzyme for other glucagon‐like peptides. Regul Pept. 1995;58(3):149‐156. 8577927 10.1016/0167-0115(95)00063-h

[55] Feng X , Gu Q , Gao G , Yuan L , Li Q , Zhang Y . The plasma levels of atrial natriuretic peptide and brain natriuretic peptide in type 2 diabetes treated with sodium‐glucose cotransporter‐2 inhibitor. Ann Endocrinol (Paris). 2020;81(5):476‐481. doi:10.1016/j.ando.2020.07.1113 32822653

[56] Frias JP , Nauck MA , Van J , et al. Efficacy and tolerability of tirzepatide, a dual glucose‐dependent insulinotropic peptide and glucagon‐like peptide‐1 receptor agonist in patients with type 2 diabetes: a 12‐week, randomized, double‐blind, placebo‐controlled study to evaluate different dose‐escalation regimens. Diabetes Obes Metab. 2020;22(6):938‐946. 31984598 10.1111/dom.13979 PMC7318331

[57] Zhao Y , Zhou Y , Xiao M , et al. Impaired glucose tolerance is associated with enhanced postprandial pancreatic polypeptide secretion. J Diabetes. 2022;14(5):334‐344. 35437937 10.1111/1753-0407.13268 PMC9366580

[58] Brereton MF , Vergari E , Zhang Q , Clark A . Alpha‐, delta‐and PP‐cells: are they the architectural cornerstones of islet structure and co‐ordination? J Histochem Cytochem. 2015;63(8):575‐591. 26216135 10.1369/0022155415583535 PMC4530398

[59] Seymour N , Brunicardi F , Chaiken R , et al. Reversal of abnormal glucose production after pancreatic resection by pancreatic polypeptide administration in man. Surgery. 1988;104(2):119‐129. 3041640

[60] Moro C . Targeting cardiac natriuretic peptides in the therapy of diabetes and obesity. Expert Opin Ther Targets. 2016;20(12):1445‐1452. 27786597 10.1080/14728222.2016.1254198

[61] Corti R , Burnett JC Jr , Rouleau JL , Ruschitzka F , Lüscher TF . Vasopeptidase inhibitors: a new therapeutic concept in cardiovascular disease? Circulation. 2001;104(15):1856‐1862. 11591626 10.1161/hc4001.097191

[62] Coué M , Moro C . Natriuretic peptide control of energy balance and glucose homeostasis. Biochimie. 2016;124:84‐91. 26037452 10.1016/j.biochi.2015.05.017

[63] Coué M , Barquissau V , Morigny P , et al. Natriuretic peptides promote glucose uptake in a cGMP‐dependent manner in human adipocytes. Sci Rep. 2018;8(1):1097. 29348496 10.1038/s41598-018-19619-0 PMC5773662

[64] Campbell DJ . Neprilysin inhibitors and bradykinin. Front Med. 2018;5:257. 10.3389/fmed.2018.00257 PMC6157573 30283782

[65] Irhuma M , Vally M . Use of angiotensin receptor–neprilysin inhibitors in heart failure: a paradigm shift. S Afr Fam Pract. 2016;58(5):60‐63.

[66] Jankowski M , Broderick TL , Gutkowska J . Oxytocin and cardioprotection in diabetes and obesity. BMC Endocr Disord. 2016;16(1):1‐9. 27268060 10.1186/s12902-016-0110-1 PMC4895973

[67] Al‐Kuraishy HM , Al‐Gareeb AI , Eldahshan OA , Batiha G . Oxytocin in diabetic Covid‐19 patients: a new perspective. Nat Prod Res. 2022;36:1‐2. 10.1080/14786419.2022.2124410 36121763

[68] Dargad RR , Prajapati MR , Dargad RR , Parekh JD . Sacubitril/valsartan: a novel angiotensin receptor‐neprilysin inhibitor. Indian Heart J. 2018;70:S102‐S110. 30122239 10.1016/j.ihj.2018.01.002 PMC6097164

[69] Poorgolizadeh E , Homayouni Moghadam F , Dormiani K , Rezaei N , Nasr‐Esfahani MH . Do neprilysin inhibitors walk the line? Heart ameliorative but brain threatening! Eur J Pharmacol. 2021;894:173851. doi:10.1016/j.ejphar.2021.173851 33422508

[70] Davidson EP , Coppey LJ , Holmes A , Yorek MA . Effect of inhibition of angiotensin converting enzyme and/or neutral endopeptidase on vascular and neural complications in high fat fed/low dose streptozotocin‐diabetic rats. Eur J Pharmacol. 2012;677(1–3):180‐187. doi:10.1016/j.ejphar.2011.12.003 22198047 PMC3268870

[71] Wewer Albrechtsen NJ , Møller A , Martinussen C , et al. Acute effects on glucose tolerance by neprilysin inhibition in patients with type 2 diabetes. Diabetes Obes Metab. 2022;24(10):2017‐2026. 35676803 10.1111/dom.14789 PMC9545540

[72] Brar GS , Barrow BM , Watson M , et al. Neprilysin is required for angiotensin‐(1‐7)'s ability to enhance insulin secretion via its proteolytic activity to generate angiotensin‐(1‐2). Diabetes. 2017;66(8):2201‐2212. doi:10.2337/db16-1318 28559246 PMC5521860

[73] Al‐Kuraishy HM , Hussien NR , Al‐Naimi MS , Al‐Buhadily AK , Al‐Gareeb AI , Lungnier C . Is ivermectin–azithromycin combination the next step for COVID‐19? Biomed Biotechnol Res J (BBRJ). 2020;4(5):101.

[74] Al‐Kuraishy HM , Al‐Gareeb AI . Effects of rosuvastatin on metabolic profile: versatility of dose‐dependent effect. J Adv Pharm Technol Res. 2019;10(1):33. 30815386 10.4103/japtr.JAPTR_330_18 PMC6383350

[75] Shao C , Zucker IH , Gao L . Angiotensin type 2 receptor in pancreatic islets of adult rats: a novel insulinotropic mediator. Am J Physiol‐Endocrinol Metabol. 2013;305(10):E1281‐E1291. 10.1152/ajpendo.00286.2013 PMC3840212 24085035

[76] He J , Yang Z , Yang H , et al. Regulation of insulin sensitivity, insulin production, and pancreatic β cell survival by angiotensin‐(1‐7) in a rat model of streptozotocin‐induced diabetes mellitus. Peptides. 2015;64:49‐54. 25576844 10.1016/j.peptides.2014.12.012

[77] Aggarwal G , Ramachandran V , Javeed N , et al. Adrenomedullin is up‐regulated in patients with pancreatic cancer and causes insulin resistance in β cells and mice. Gastroenterology. 2012;143(6):1510‐1517. e1. 22960655 10.1053/j.gastro.2012.08.044 PMC3787599

[78] Wong HK , Tang F , Cheung TT , Cheung BMY . Adrenomedullin and diabetes. World J Diabetes. 2014;5(3):364. 24936257 10.4239/wjd.v5.i3.364 PMC4058740

[79] Kjeldsen SA , Hansen LH , Esser N , et al. Neprilysin inhibition increases glucagon levels in humans and mice with potential effects on amino acid metabolism. J Endocr Soc. 2021;5(9):bvab084. 34337276 10.1210/jendso/bvab084 PMC8317634

[80] Packer M . Does neprilysin inhibition potentiate or minimize the adverse effects of glucagon‐like peptide‐1 receptor agonists in chronic heart failure? J Card Fail. 2018;24(2):109‐111. 29305970 10.1016/j.cardfail.2017.12.007

[81] Aston‐Mourney K , Hull RL , Zraika S , Udayasankar J , Subramanian SL , Kahn SE . Exendin‐4 increases islet amyloid deposition but offsets the resultant beta cell toxicity in human islet amyloid polypeptide transgenic mouse islets. Diabetologia. 2011;54(7):1756‐1765. doi:10.1007/s00125-011-2143-3 21484213 PMC3220951

[82] Elshaer F , Lawand S , Mohamed Z , Al Ayoubi F , Hanfi Y , AlQarni A . Efficacy and safety outcome of angiotensin receptor‐Neprilysin inhibitors (ARNIs) in patients with heart failure and preserved ejection fraction (HFpEF): preliminary results. Res Rep Clin Cardiol. 2020;11:39‐47.

